# Practicing Histotechnologists Identify the Core Competencies Needed by Newly Graduated Biomedical Laboratory Scientists in Histotechnology and Histology

**DOI:** 10.1007/s40670-019-00770-w

**Published:** 2019-07-16

**Authors:** Eeva Liikanen

**Affiliations:** Tampere University of Applied Sciences, Tampere, Finland

**Keywords:** Biomedical laboratory scientist, Core competencies, Curricula, Histotechnologist, Histotechnology

## Abstract

The universities of applied sciences in Finland offer 3.5-year courses for histotechnologists and they graduate as biomedical laboratory scientist with 12 credits in histology and histotechnology. The aim of this study was to survey practicing histotechnologists about the core competencies needed by newly graduated biomedical scientists in histology and histotechnology. The data were collected in Finland in 2015. We asked 43 participants to complete a questionnaire that comprised two background questions, five open-ended questions and 38 Likert scale questions, with the responses ranging from five (strongly agree) to one (strongly disagree), and 22 (51%) responded. They stated that the most important competencies were the principles of tissue processing (mean 4.77), embedding (4.64), laboratory safety (4.57), fixation methods (4.55), cutting by microtomy (4.55), quality control of sections (4.55), fixation methods (4.55), and principles of stains (4.36). The least important competencies were quality control of molecular pathology (2.56), interpretation of immunohistological stains (2.71), use of molecular pathology (2.89), and independent dissection (2.91). The respondents stated that there were 20 stains that newly graduated biomedical laboratory scientists needed to know. The practices involving staining emerged in the open responses and four were considered to be important: Hematoxylin-Eosin (*n* = 18), Periodic Acid Schiff (*n =* 11), Alcian Blue-Periodic Acid Schiff (*n =* 9), and Giemsa (*n =* 9). The most essential tissues to identify were the histology of the alimentary track (*n =* 9), skin (*n =* 6), and liver (*n =* 5). The core competencies that histotechnologists felt were important for newly graduated biomedical laboratory scientists seemed to be consistent with the current curriculum.

## Introduction

In Finland, histotechnologists are educated in universities of applied sciences and they graduate with a bachelor’s degree in biomedical laboratory science if they achieve 210 European Credit Transfer and Accumulation System credits after 3.5 years. Once they have acquired their degree, biomedical scientists are able to work in all kinds of clinical laboratories, such as those covering clinical chemistry, histology, hematology, and physiology. There is no existing national curriculum in Finland, but the courses that are provided are very similar and they all offer 12 credits in histology and histotechnology studies, providing graduates with basic competencies in histology and histotechnology.

The incidence of new cancer cases is continuing to rise in Finland [1] and the situation is same in other countries. Oncology specimens are increasingly being investigated in histology laboratories and new methods are being developed. The aim of the universities of applied science is to provide professionals with the theoretical knowledge and practical expertise they need for their working life and this is backed up by Finnish legislation [2]. The curricula covering degrees for biomedical laboratory science include basic histology and histotechnology, but they all need to be reviewed and evaluated to ensure that they prepare graduates for a challenging and fast-changing working environment. Basic histology covers the structure of common tissues. Histotechnology consists of the basic histological processes, from specimen types to quality control, and it includes staining and frozen sections processes, laboratory safety and immunohistochemistry, excluding interpreting immunohistological stains. The curricula also include molecular pathology, but only in terms of theoretical instruction.

There have only been a few studies about competencies in histotechnology. Almost 20 years ago, research was published that stated that histotechnology students needed to develop their competencies in immunohistochemistry, laboratory information systems, immunofluorescence, waste management, and quality assurance in in situ nucleic hybridization [3]. Since then, skills in diagnostic techniques, such as molecular diagnostics, have proved to be essential for laboratory professionals [4]. Unfortunately, the educational curricula for molecular diagnostics are not well defined [5] and they need to be assessed to ensure that they are integrated into the basic professional education that is provided.

There have been a few studies about the curricula offered to biomedical laboratory scientists [6, 7], but they have not focused on histology or histotechnology. Some studies about histology and medical students [8–10] have been published and research has shown that medical students are more motivated to learn about histology than dentistry and pharmacy [11].

## Aim

The aim of this study was to survey practicing histotechnologists about the core competencies needed by newly graduated biomedical scientists in histology and histotechnology. We hope that these findings can be used to help develop curricula in biomedical laboratory science programs in Finland. They may also provide useful feedback for countries with similar healthcare systems.

## Materials and Method

The questions in the questionnaire were based on the curricula of the degree programs offered by the Finnish universities of applied sciences and they included basic histotechnology and histology. There are currently about 300 practicing histotechnologists in Finland and the survey data were collected by surveying histotechnologists who were attending a national histotechnological symposium in Finland in 2015. The event was organized by the Board of the Finnish Association of Histotechnology and aimed at histotechnologists who worked in different histology laboratories around Finland. We asked 43 participants to complete the questionnaire and 22 (51%) responded. There were two background questions that covered where the participants worked and their experience of histotechnology, five open-ended questions and 38 Likert scale questions, with responses ranging from five for strongly agree to one for strongly disagree. The Likert scale questions that covered the participants’ competencies were divided to eight sections: 18 questions on specimens and tissue processing, six on staining, two on frozen sections, five on immunohistochemistry, three on molecular pathology, two on covering, and one on the structure of tissues and laboratory safety.

The open-ended questions asked the participants to list the stainings, frozen section stainings, and histological tissue structures that newly graduated biomedical laboratory scientist should know. They also explored what newly graduated biomedical laboratory scientist should know about molecular pathology.

The data were collected at the event with the permission of the Board of the Finnish Association of Histotechnology and analyzed by using SPSS version 23.0 (IBM Corp, New York, USA). Responding to the questionnaire was voluntary and completing it provided written, informed consent for the data to be used.

## Results

The 22 histotechnologists who responded had an average of 16.9 years (range 2–40 years) of relevant work experience and came from different geographical locations in Finland: 10 were from the north, six from the east, three from the south, two from the west, and one from the middle of Finland.

The respondents were asked to identify the most important competencies for histotechnologists and scored them on the 5-point Likert scale, with a higher value indicating greater importance (Table 1). The responses and mean scores were as follows: principles of tissue processing (4.77), embedding (4.64), laboratory safety (4.57), fixation methods (4.55), cutting by microtomy (4.55), quality control of sections (4.55), fixation methods (4.55), principles of stains (4.36), artifacts of fixation (4.32), and staining by hand (4.23). The least important competencies were as follows: quality control of molecular pathology (2.56), interpretation of immunohistological stains (2.71), use of molecular pathology (2.89), dissecting independently (2.91), and using immunohistochemistry stainers (2.95). Laboratory safety was also considered important and was joint third on the list (4.57).

**Table 1:** Mean and standard deviation (SD) scores given by the 22 histotechnologists for the various competencies, including the number of responses to each question

Competence	Mean	SD	Responses
Specimens and tissue processing			
Specimen types	3.91	0.92	22
Specimen collection	3.23	0.86	22
Name of investigation	3.41	0.85	22
Artifacts of fixation and preventing them	4.32	0.83	22
Fixation methods	4.55	0.51	22
Requesting information on specimen	4.18	0.91	22
Specimen reception and recording	4.00	0.92	22
Grossing small specimens	4.00	1.02	22
Grossing independently (e.g., gall bladder, skin)	2.91	1.101	22
Theory of grossing (e.g., gall bladder, skin)	3.45	1.01	22
Assist a pathologist in grossing	3.95	0.76	22
Principles of tissue processing	4.77	0.51	22
Use of tissue processor	4.00	0.87	22
Maintaining tissue processor	3.64	1.05	22
Quality assurance of tissue processing	4.00	0.98	22
Embedding	4.64	0.66	22
Cutting with a microtome	4.55	0.80	22
Quality control of sections	4.55	0.67	22
Staining			
Principles of staining	4.36	0.73	22
Staining by hand	4.23	0.87	22
Use of automated stainer	4.09	0.87	22
Clinical indications of stains	4.00	0.87	22
Interpreting stains	3.41	0.91	22
Quality control of staining	4.18	0.85	22
Frozen sections			
Preparing frozen sections	3.95	0.86	21
Staining of frozen sections	3.90	0.77	21
Immunohistochemistry			
Principles of immunohistochemistry	4.05	0.97	21
Clinical indications of immunohistochemistry	3.48	0.75	21
Use of immunohistological stainer	2.95	1.16	21
Interpreting immunohistological stains	2.71	0.90	21
Quality control of immunohistological stains	3.29	1.10	21
Molecular pathology			
Principles of molecular pathology methods	3.22	1.11	18
Use of molecular pathology	2.89	0.96	18
Quality control of molecular pathology	2.56	0.92	18
Histological structure of tissues	3.60	1.05	20
Covering			
Manual cover slipping	3.90	1.14	21
Automatic cover slipping	4.00	0.89	21
Laboratory safety	4.57	0.68	21

The respondents listed the 20 stains that newly graduated biomedical laboratory scientists should know. The four stains that were considered the most important were as follows: Hematoxylin-Eosin (*n =* 18), Periodic Acid Schiff (*n =* 11), Alcian Blue-Periodic Acid Schiff (*n =* 9), and Giemsa (*n =* 9). The respondents also stated the most essential tissues that scientists should be able to identify and these were the alimentary track (*n =* 9), skin (*n =* 6) and liver (*n =* 5). Only one respondent listed the breast and prostate. One respondent listed the Hematoxylin-Eosin stain when asked about frozen sections. Two respondents considered principles of molecular biology under important competencies and one respondent mentioned basic methods. In addition, one respondent each reported that teamwork, ergonomics, and the responsibilities and rights of the employee were important competencies for newly graduated biomedical laboratory scientists.

## Discussion

It important to review the curricula offered to healthcare students at regular intervals. Curriculum mapping can be an essential tool for developing, reviewing, improving, and refining any integrated curriculum however complex [12]. This study reviewed the curricula offered to biomedical laboratory scientists who studied histotechnology and histology at universities of applied sciences in Finland from the histotechnologists’ point of view. In 2014, all of the histotechnology and histology degree programs offered by the country’s six universities of applied sciences provided curricula that covered the same competencies, the same basic study contents, and the same number of professional credits. Although there is no national curriculum for biomedical scientists, the contents of the curricula offered by different institutions are quite similar. Educators are responsible for developing curricula, but it is advisable that they take note of the feedback from graduates who have undergone these courses when they put the new skills and knowledge they have learnt into practice.

The curricula in health and medical education primarily focus on evidence-based knowledge. However, when they are being developed, they also need to pay attention to the views of professionals, including physicians, nurses, and other health professionals, as they are best placed to know what kind of competencies are required in practice.

The histotechnologists who responded to our survey told us that specimen and tissue processing, staining, and laboratory safety were very important and they particularly mentioned fixation methods, principles of tissue processing, embedding, cutting with a microtome and quality control of sections in specimens and tissue processing. They also emphasized the importance of the principles of staining and staining by hand. These are competencies that have been relevant to this role for almost a century and it is remarkable that they still are today.

Immunohistochemistry has been used for decades and its use is set to increase in the future. Histotechnologists stated that the principles and clinical indications of immunohistochemistry were important in immunohistochemistry, but they did not feel that they were important competencies for newly graduated histotechnologists. That was because the interpretation of immunohistological stains is the responsibility of pathologists in Finland and carrying out quality assessments of immunohistological stains falls under the remit of cell biologists. Although molecular diagnostics is regarded as essential for laboratory professionals [4], the feedback from this study was that it was not considered very important for newly graduated histotechnologists. In fact, very few histotechnologists used molecular diagnostics in their work in Finland.

The histological structure of tissues was considered quite important by our respondents and the most essential tissues were the alimentary track, skin, and liver. Traditionally, students have learnt tissue structures by looking at slides with microscopes, but these days it is possible to use virtual slides and they offer excellent possibilities for learning [13, 14]. Virtual microscopes have been reported to be as good as optical microscopy when it comes to learning histology [15] and their use has increased in education [16]. Digitalization also offers diverse possibilities for learning histology and two developments in this field are a three-dimensional, interactive online resource on the epithelium [17] and a virtual microscope histology laboratory for learning histotechnology [18].

The small number of respondents could be seen as a limitation of this study, but their everyday work experiences provided valuable feedback on the core competencies that are needed in histotechnology and histology. Working life is a good way of highlighting the competencies that are needed in a particular role, but we also acknowledge that some post holders can become stuck in their ways and not respond to changing needs and new technology. It could also be useful to ask pathologists and cell biologists to complete similar surveys, because they can provide a wider overall view of histotechnology and its future applications.

## Conclusions

The responses to our questionnaire indicated that the core competencies that were considered to be important for newly graduated histotechnologists in Finland seemed to be consistent with the curricula offered to biomedical laboratory scientists by the country’s universities of applied science.
